# Supplementary material for: Phenotypic plasticity as a mechanism of cave colonization and adaptation
Source: eLife. 2020 Apr 21;9:e51830. doi: 10.7554/eLife.51830 (PMC7173965; doi:10.7554/eLife.51830)
Supplement: Figure 2—source data 1. [file elife-51830-fig2-data1.docx]

Figure 2 – Source Data 1. List of differentially expressed genes (DEG) between three surface fish (7 month-old) placed in either totally dark (D/D) or light/ dark (L/D) conditions within 1 day post fertilization. Differential expression was determined using the Cufflinks pipeline. Significance threshold was set at p_adj_<0.1.

| Locus | gene_short_name | Dark(FPKM) | Light(FPKM) | Light_Dark_p_value | Light_Dark_q_value | Light_Dark_value_1 | Light_Dark_value_2 | Light_Dark_log2_fold_change | Light_Dark_test_stat | length |
| --- | --- | --- | --- | --- | --- | --- | --- | --- | --- | --- |
| XLOC_000025 | sytl5 | 1.72938 | 4.90491 | 1e-04 | 0.0124776 | 1.72938 | 4.90491 | 1.50397 | 2.20332 | KB871578.1:1440714-1462997 |
| XLOC_000093 | inhbb | 2.81404 | 10.2465 | 5e-05 | 0.00738566 | 2.81404 | 10.2465 | 1.86442 | 2.82349 | KB871578.1:7510497-7518815 |
| XLOC_000111 | gpr143 | 2.81896 | 0.994148 | 0.0013 | 0.0811047 | 2.81896 | 0.994148 | -1.50363 | -1.92644 | KB871578.1:492654-518016 |
| XLOC_000262 | rag1 | 0.888883 | 3.23342 | 0.00045 | 0.0403574 | 0.888883 | 3.23342 | 1.86299 | 2.17165 | KB871579.1:5211102-5217994 |
| XLOC_000295 | nr2e3 | 6.71822 | 1.71059 | 5e-05 | 0.00738566 | 6.71822 | 1.71059 | -1.97358 | -2.89312 | KB871579.1:8814852-8819179 |
| XLOC_000399 | actc1c | 23.6314 | 9.25617 | 0.00075 | 0.0540027 | 23.6314 | 9.25617 | -1.35222 | -1.98907 | KB871580.1:16121-28370 |
| XLOC_000433 | asb12b | 8.43801 | 3.26271 | 0.00085 | 0.0574938 | 8.43801 | 3.26271 | -1.37083 | -1.95768 | KB871580.1:3001595-3006229 |
| XLOC_000452 | zgc:174917 | 8.95196 | 57.3576 | 5e-05 | 0.00738566 | 8.95196 | 57.3576 | 2.67971 | 3.59156 | KB871580.1:5079647-5085941 |
| XLOC_000453 | si:ch211-153b23.4 | 11.8002 | 73.0788 | 5e-05 | 0.00738566 | 11.8002 | 73.0788 | 2.63065 | 3.34646 | KB871580.1:5095627-5104246 |
| XLOC_000454 | irg1l | 22.5864 | 132.136 | 5e-05 | 0.00738566 | 22.5864 | 132.136 | 2.54849 | 2.91901 | KB871580.1:5113417-5116993 |
| XLOC_000485 | dyrk4 | 11.7941 | 3.88336 | 5e-05 | 0.00738566 | 11.7941 | 3.88336 | -1.60269 | -2.4286 | KB871580.1:2111547-2129387 |
| XLOC_000515 | si:ch211-153b23.5 | 157.673 | 712.965 | 0.00055 | 0.0450978 | 157.673 | 712.965 | 2.1769 | 2.27869 | KB871580.1:5088174-5094399 |
| XLOC_000580 | golt1a | 12.1484 | 3.97679 | 5e-05 | 0.00738566 | 12.1484 | 3.97679 | -1.6111 | -2.3119 | KB871584.1:999779-1004564 |
| XLOC_000649 | rbp2b | 2.60755 | 0.53257 | 0.001 | 0.0665199 | 2.60755 | 0.53257 | -2.29165 | -2.18177 | KB871588.1:322775-327198 |
| XLOC_000702 | ENSAMXG00000016396 | 15.7313 | 6.56485 | 0.0015 | 0.0878528 | 15.7313 | 6.56485 | -1.2608 | -1.87407 | KB871590.1:710199-717388 |
| XLOC_000735 | ENSAMXG00000002944 | 0.635797 | 3.46134 | 7e-04 | 0.0518904 | 0.635797 | 3.46134 | 2.44469 | 2.15452 | KB871592.1:79745-81396 |
| XLOC_000771 | mpx | 17.9612 | 58.2451 | 5e-05 | 0.00738566 | 17.9612 | 58.2451 | 1.69725 | 2.58898 | KB871593.1:122277-151875 |
| XLOC_000852 | ftcd | 8.39321 | 3.25075 | 5e-05 | 0.00738566 | 8.39321 | 3.25075 | -1.36845 | -2.12144 | KB871596.1:296241-307344 |
| XLOC_000916 | ENSAMXG00000008669 | 2.28762 | 8.78731 | 0.00055 | 0.0450978 | 2.28762 | 8.78731 | 1.94158 | 2.31209 | KB871598.1:979360-1015819 |
| XLOC_000921 | tecra | 19.2998 | 8.25754 | 0.00155 | 0.0902549 | 19.2998 | 8.25754 | -1.2248 | -1.89801 | KB871599.1:37705-47301 |
| XLOC_000952 | gcgra | 1.08799 | 2.77062 | 0.00125 | 0.0789662 | 1.08799 | 2.77062 | 1.34855 | 1.89347 | KB871599.1:242400-262385 |
| XLOC_000970 | soul5 | 9.69748 | 44.2789 | 5e-05 | 0.00738566 | 9.69748 | 44.2789 | 2.19094 | 2.8049 | KB871599.1:974982-1005025 |
| XLOC_000984 | unc45b | 29.1538 | 8.21331 | 5e-05 | 0.00738566 | 29.1538 | 8.21331 | -1.82765 | -2.7806 | KB871601.1:371258-381174 |
| XLOC_001030 | ENSAMXG00000013132 | 3.5872 | 1.23813 | 0.00145 | 0.0861806 | 3.5872 | 1.23813 | -1.53469 | -1.91854 | KB871601.1:1187756-1192073 |
| XLOC_001093 | si:ch211-244a23.1 | 4.09304 | 1.05886 | 5e-05 | 0.00738566 | 4.09304 | 1.05886 | -1.95066 | -2.68567 | KB871603.1:346775-425210 |
| XLOC_001264 | nr4a3 | 1.48495 | 3.71457 | 5e-04 | 0.0432953 | 1.48495 | 3.71457 | 1.32278 | 2.02312 | KB871610.1:518753-561832 |
| XLOC_001293 | rln3b | 3.74295 | 0.853494 | 0.00075 | 0.0540027 | 3.74295 | 0.853494 | -2.13272 | -2.49295 | KB871612.1:143776-147078 |
| XLOC_001328 | crtac1a | 2.13533 | 11.401 | 5e-05 | 0.00738566 | 2.13533 | 11.401 | 2.41662 | 3.5946 | KB871612.1:859389-870145 |
| XLOC_001370 | pmela | 8.66005 | 3.77339 | 0.0012 | 0.0770185 | 8.66005 | 3.77339 | -1.19852 | -1.86439 | KB871614.1:1074424-1091771 |
| XLOC_001576 | itga6l | 6.81959 | 33.3774 | 5e-05 | 0.00738566 | 6.81959 | 33.3774 | 2.29111 | 3.2917 | KB871621.1:913994-946351 |
| XLOC_001598 | tlr5a | 1.58578 | 4.00942 | 0.00145 | 0.0861806 | 1.58578 | 4.00942 | 1.3382 | 1.90087 | KB871623.1:354083-356732 |
| XLOC_001630 | ENSAMXG00000012407 | 7.88452 | 0.972725 | 5e-05 | 0.00738566 | 7.88452 | 0.972725 | -3.01892 | -3.58831 | KB871624.1:632513-689013 |
| XLOC_001660 | ENSAMXG00000015910 | 0.677565 | 3.24349 | 5e-05 | 0.00738566 | 0.677565 | 3.24349 | 2.25912 | 2.56101 | KB871626.1:91983-98799 |
| XLOC_001783 | ENSAMXG00000021817 | 4.54948 | 1.27881 | 2e-04 | 0.0216011 | 4.54948 | 1.27881 | -1.8309 | -2.3516 | KB871630.1:5861922-5865492 |
| XLOC_001824 | tyrp1b | 1.50872 | 0.276749 | 2e-04 | 0.0216011 | 1.50872 | 0.276749 | -2.44668 | -2.7465 | KB871630.1:5217534-5227365 |
| XLOC_001840 | fgg | 61.5504 | 21.1568 | 3e-04 | 0.0296882 | 61.5504 | 21.1568 | -1.54065 | -2.08075 | KB871630.1:5844873-5851876 |
| XLOC_001917 | ENSAMXG00000028266 | 32.4304 | 10.5147 | 5e-05 | 0.00738566 | 32.4304 | 10.5147 | -1.62494 | -2.56256 | KB871633.1:1064227-1073679 |
| XLOC_001944 | ENSAMXG00000003102 | 90.7779 | 790.256 | 5e-05 | 0.00738566 | 90.7779 | 790.256 | 3.12191 | 3.2252 | KB871635.1:35154-42471 |
| XLOC_001955 | obsl1b | 7.83515 | 3.47243 | 0.00115 | 0.0742841 | 7.83515 | 3.47243 | -1.17401 | -1.81193 | KB871635.1:731204-770758 |
| XLOC_001973 | lipca | 2.1924 | 0.167918 | 0.00105 | 0.0689328 | 2.1924 | 0.167918 | -3.70668 | -2.88502 | KB871636.1:533700-560666 |
| XLOC_002044 | si:ch211-81a5.8 | 6.41447 | 0.866872 | 5e-05 | 0.00738566 | 6.41447 | 0.866872 | -2.88744 | -3.42552 | KB871640.1:869861-877544 |
| XLOC_002047 | ENSAMXG00000010135 | 87.6827 | 22.9572 | 5e-05 | 0.00738566 | 87.6827 | 22.9572 | -1.93334 | -2.52617 | KB871640.1:1055303-1099497 |
| XLOC_002080 | cuzd1.2 | 64.7338 | 19.8308 | 5e-05 | 0.00738566 | 64.7338 | 19.8308 | -1.70678 | -2.69252 | KB871643.1:701902-721166 |
| XLOC_002086 | htra1a | 7.4593 | 3.14847 | 0.00075 | 0.0540027 | 7.4593 | 3.14847 | -1.24439 | -1.95411 | KB871643.1:547526-613060 |
| XLOC_002112 | ENSAMXG00000004676 | 56.1736 | 25.2781 | 0.0013 | 0.0811047 | 56.1736 | 25.2781 | -1.15201 | -1.89297 | KB871644.1:889063-897015 |
| XLOC_002320 | ENSAMXG00000009532 | 6.6181 | 16.2928 | 8e-04 | 0.0556097 | 6.6181 | 16.2928 | 1.29975 | 1.99253 | KB871654.1:944058-952616 |
| XLOC_002453 | ENSAMXG00000001841 | 8.72091 | 3.03604 | 0.00075 | 0.0540027 | 8.72091 | 3.03604 | -1.52229 | -2.00856 | KB871662.1:842321-847168 |
| XLOC_002460 | apoa2 | 1446.47 | 417.417 | 2e-04 | 0.0216011 | 1446.47 | 417.417 | -1.79297 | -2.10386 | KB871662.1:755634-759753 |
| XLOC_002461 | apoc4 | 13.0281 | 4.64507 | 0.0016 | 0.0923632 | 13.0281 | 4.64507 | -1.48786 | -1.95224 | KB871662.1:763921-766276 |
| XLOC_002528 | ENSAMXG00000015132 | 11.122 | 32.2114 | 5e-05 | 0.00738566 | 11.122 | 32.2114 | 1.53415 | 2.31617 | KB871666.1:617191-660944 |
| XLOC_002544 | ENSAMXG00000018298 | 1.48336 | 0 | 5e-05 | 0.00738566 | 1.48336 | 0 | -Inf | NA | KB871667.1:715875-729135 |
| XLOC_002557 | casq1b | 39.448 | 12.6542 | 1e-04 | 0.0124776 | 39.448 | 12.6542 | -1.64034 | -2.51524 | KB871667.1:596086-630314 |
| XLOC_002592 | pde6ha | 153.962 | 990.1 | 5e-05 | 0.00738566 | 153.962 | 990.1 | 2.685 | 4.25525 | KB871670.1:579803-580207 |
| XLOC_002599 | si:zfos-323e3.4 | 2.33197 | 6.29524 | 8e-04 | 0.0556097 | 2.33197 | 6.29524 | 1.43271 | 2.09449 | KB871670.1:218416-232095 |
| XLOC_002665 | ENSAMXG00000007037 | 1524.76 | 509.863 | 4e-04 | 0.0372019 | 1524.76 | 509.863 | -1.5804 | -2.06446 | KB871673.1:828202-829527 |
| XLOC_002845 | ENSAMXG00000015374 | 26.3848 | 11.1454 | 0.00085 | 0.0574938 | 26.3848 | 11.1454 | -1.24326 | -1.92956 | KB871682.1:405661-408875 |
| XLOC_002905 | ENSAMXG00000017046 | 18.7434 | 101.724 | 5e-05 | 0.00738566 | 18.7434 | 101.724 | 2.44021 | 2.84071 | KB871685.1:170515-179715 |
| XLOC_003111 | ENSAMXG00000007743 | 489.302 | 3068.49 | 5e-05 | 0.00738566 | 489.302 | 3068.49 | 2.64873 | 2.73331 | KB871688.1:5644019-5646112 |
| XLOC_003118 | nt5c2l1 | 1.31007 | 3.22673 | 0.00085 | 0.0574938 | 1.31007 | 3.22673 | 1.30043 | 1.91801 | KB871689.1:45593-61053 |
| XLOC_003124 | slc5a1 | 13.5913 | 47.7812 | 5e-05 | 0.00738566 | 13.5913 | 47.7812 | 1.81376 | 2.89548 | KB871689.1:411283-432176 |
| XLOC_003129 | ENSAMXG00000028718 | 15.9252 | 63.1131 | 5e-05 | 0.00738566 | 15.9252 | 63.1131 | 1.98663 | 3.07609 | KB871689.1:749657-763337 |
| XLOC_003159 | aglb | 8.2341 | 3.12566 | 1e-04 | 0.0124776 | 8.2341 | 3.12566 | -1.39745 | -2.25824 | KB871691.1:425429-466515 |
| XLOC_003208 | ENSAMXG00000014187 | 26.4581 | 7.27278 | 5e-05 | 0.00738566 | 26.4581 | 7.27278 | -1.86313 | -2.39019 | KB871694.1:716826-718209 |
| XLOC_003273 | srl | 14.8543 | 5.28934 | 5e-05 | 0.00738566 | 14.8543 | 5.28934 | -1.48972 | -2.33184 | KB871702.1:150751-170936 |
| XLOC_003307 | nr1d1 | 13.821 | 5.389 | 5e-05 | 0.00738566 | 13.821 | 5.389 | -1.35877 | -2.20071 | KB871704.1:426428-435796 |
| XLOC_003375 | ENSAMXG00000014747 | 73.4764 | 26.5773 | 0.00085 | 0.0574938 | 73.4764 | 26.5773 | -1.46709 | -1.98389 | KB871707.1:35060-44134 |
| XLOC_003531 | cyp8b2 | 17.2369 | 5.96801 | 5e-05 | 0.00738566 | 17.2369 | 5.96801 | -1.53018 | -2.28808 | KB871717.1:704847-706458 |
| XLOC_003553 | ENSAMXG00000014372 | 4.50593 | 1.48769 | 5e-04 | 0.0432953 | 4.50593 | 1.48769 | -1.59874 | -2.1852 | KB871718.1:576394-607652 |
| XLOC_003924 | mybpha | 39.8948 | 14.4617 | 0.00015 | 0.0178305 | 39.8948 | 14.4617 | -1.46396 | -2.36596 | KB871745.1:680467-705649 |
| XLOC_003980 | maats1 | 1.25126 | 0.439702 | 0.00125 | 0.0789662 | 1.25126 | 0.439702 | -1.50878 | -1.95201 | KB871750.1:479109-515783 |
| XLOC_003987 | gpx7 | 5.53336 | 1.77059 | 1e-04 | 0.0124776 | 5.53336 | 1.77059 | -1.64392 | -2.22967 | KB871751.1:456402-470647 |
| XLOC_004077 | uox | 25.9104 | 8.36595 | 5e-05 | 0.00738566 | 25.9104 | 8.36595 | -1.63093 | -2.36451 | KB871753.1:1074353-1080629 |
| XLOC_004096 | ENSAMXG00000017658 | 25.8306 | 8.66116 | 0.00045 | 0.0403574 | 25.8306 | 8.66116 | -1.57645 | -2.08683 | KB871753.1:2178555-2200020 |
| XLOC_004174 | tgm2a | 17.5466 | 3.81582 | 5e-05 | 0.00738566 | 17.5466 | 3.81582 | -2.20113 | -3.14917 | KB871754.1:5123302-5130631 |
| XLOC_004261 | sst1.2 | 15.8065 | 109.086 | 5e-05 | 0.00738566 | 15.8065 | 109.086 | 2.78688 | 3.1029 | KB871758.1:283368-286701 |
| XLOC_004289 | ENSAMXG00000002805 | 5.04307 | 28.0732 | 5e-05 | 0.00738566 | 5.04307 | 28.0732 | 2.47682 | 3.45563 | KB871761.1:16057-18860 |
| XLOC_004290 | ENSAMXG00000002840 | 0.400355 | 2.39441 | 3e-04 | 0.0296882 | 0.400355 | 2.39441 | 2.58032 | 2.6615 | KB871761.1:23088-33199 |
| XLOC_004309 | rbp3 | 19.536 | 55.9164 | 5e-05 | 0.00738566 | 19.536 | 55.9164 | 1.51714 | 2.42609 | KB871761.1:382801-385067 |
| XLOC_004337 | ENSAMXG00000018234 | 6.72252 | 2.51078 | 0.00055 | 0.0450978 | 6.72252 | 2.51078 | -1.42087 | -2.00482 | KB871763.1:444998-472622 |
| XLOC_004372 | col10a1a | 125.49 | 41.3909 | 1e-04 | 0.0124776 | 125.49 | 41.3909 | -1.60019 | -2.24698 | KB871767.1:433097-441907 |
| XLOC_004484 | txn | 165.002 | 359.659 | 0.0015 | 0.0878528 | 165.002 | 359.659 | 1.12415 | 1.79726 | KB871773.1:351811-358272 |
| XLOC_004868 | ENSAMXG00000004646 | 4.34645 | 1.54864 | 0.00055 | 0.0450978 | 4.34645 | 1.54864 | -1.48883 | -2.0516 | KB871797.1:262399-274704 |
| XLOC_004873 | tm4sf4 | 530.68 | 162.437 | 0.00025 | 0.0254937 | 530.68 | 162.437 | -1.70797 | -2.29356 | KB871797.1:586846-596495 |
| XLOC_004879 | ENSAMXG00000016751 | 2.14144 | 5.73489 | 0.00025 | 0.0254937 | 2.14144 | 5.73489 | 1.42118 | 2.09893 | KB871798.1:173791-194682 |
| XLOC_004908 | ENSAMXG00000013747 | 1.84443 | 7.24206 | 5e-04 | 0.0432953 | 1.84443 | 7.24206 | 1.97322 | 2.24199 | KB871800.1:304609-308211 |
| XLOC_004996 | ENSAMXG00000013876 | 1.5781 | 9.6664 | 2e-04 | 0.0216011 | 1.5781 | 9.6664 | 2.61479 | 2.46262 | KB871804.1:568886-571789 |
| XLOC_005033 | ENSAMXG00000007580 | 7.63171 | 2.40503 | 5e-05 | 0.00738566 | 7.63171 | 2.40503 | -1.66595 | -2.48256 | KB871807.1:217452-235079 |
| XLOC_005217 | fabp10a | 513.26 | 122.233 | 5e-05 | 0.00738566 | 513.26 | 122.233 | -2.07005 | -2.62509 | KB871824.1:186324-193248 |
| XLOC_005233 | rorca | 3.93054 | 10.2191 | 2e-04 | 0.0216011 | 3.93054 | 10.2191 | 1.37847 | 2.07223 | KB871825.1:273511-307601 |
| XLOC_005239 | nexn | 17.6518 | 7.06033 | 6e-04 | 0.0484072 | 17.6518 | 7.06033 | -1.32201 | -2.03959 | KB871825.1:551365-582477 |
| XLOC_005272 | cps1 | 3.07537 | 1.26253 | 0.00055 | 0.0450978 | 3.07537 | 1.26253 | -1.28445 | -1.87905 | KB871827.1:115446-246773 |
| XLOC_005342 | hmox1a | 1.63559 | 7.73547 | 5e-05 | 0.00738566 | 1.63559 | 7.73547 | 2.24168 | 2.75381 | KB871831.1:280111-282576 |
| XLOC_005499 | fads2 | 142.981 | 36.8543 | 5e-05 | 0.00738566 | 142.981 | 36.8543 | -1.95592 | -2.92632 | KB871834.1:3460641-3478702 |
| XLOC_005516 | hal | 9.97762 | 3.83312 | 5e-04 | 0.0432953 | 9.97762 | 3.83312 | -1.38018 | -2.01239 | KB871834.1:4355479-4371595 |
| XLOC_005520 | tph1a | 13.4651 | 4.63457 | 1e-04 | 0.0124776 | 13.4651 | 4.63457 | -1.53872 | -2.27977 | KB871834.1:4504607-4511760 |
| XLOC_005569 | ENSAMXG00000004797 | 267.793 | 80.7565 | 0.00025 | 0.0254937 | 267.793 | 80.7565 | -1.72947 | -2.10168 | KB871837.1:315052-319078 |
| XLOC_005599 | clul1 | 5.57972 | 12.8579 | 0.00145 | 0.0861806 | 5.57972 | 12.8579 | 1.20439 | 1.82359 | KB871840.1:280159-290652 |
| XLOC_005798 | per2 | 2.00363 | 6.60999 | 5e-05 | 0.00738566 | 2.00363 | 6.60999 | 1.72203 | 2.71497 | KB871856.1:269900-309108 |
| XLOC_005825 | gpbar1 | 1.19921 | 3.67477 | 0.0014 | 0.0852261 | 1.19921 | 3.67477 | 1.61557 | 1.97244 | KB871858.1:599602-600616 |
| XLOC_005958 | nr1d4a | 3.49854 | 1.3138 | 0.00105 | 0.0689328 | 3.49854 | 1.3138 | -1.41301 | -1.89739 | KB871870.1:467456-493980 |
| XLOC_005996 | slc39a8 | 11.5426 | 4.6383 | 9e-04 | 0.0606715 | 11.5426 | 4.6383 | -1.31531 | -1.86312 | KB871873.1:449404-454954 |
| XLOC_006012 | nmbb | 2.41993 | 0 | 5e-05 | 0.00738566 | 2.41993 | 0 | -Inf | NA | KB871875.1:56-661 |
| XLOC_006122 | zgc:85843 | 24.0139 | 8.05679 | 1e-04 | 0.0124776 | 24.0139 | 8.05679 | -1.57559 | -2.25041 | KB871883.1:60492-87189 |
| XLOC_006155 | ENSAMXG00000012871 | 16.6802 | 5.64746 | 1e-04 | 0.0124776 | 16.6802 | 5.64746 | -1.56246 | -2.43038 | KB871886.1:202267-238321 |
| XLOC_006189 | kdm6bb | 7.10864 | 2.55495 | 0.0016 | 0.0923632 | 7.10864 | 2.55495 | -1.47628 | -1.96655 | KB871888.1:152503-181265 |
| XLOC_006192 | hhatla | 58.8582 | 23.0381 | 4e-04 | 0.0372019 | 58.8582 | 23.0381 | -1.35322 | -2.11597 | KB871889.1:2464-18923 |
| XLOC_006425 | ENSAMXG00000012617 | 23.952 | 8.70033 | 2e-04 | 0.0216011 | 23.952 | 8.70033 | -1.461 | -2.09588 | KB871909.1:523073-526998 |
| XLOC_006426 | ENSAMXG00000012633 | 19.7979 | 7.31712 | 0.00065 | 0.0502225 | 19.7979 | 7.31712 | -1.436 | -1.9465 | KB871909.1:531694-533828 |
| XLOC_006545 | krtt1c19e | 24.6566 | 8.80509 | 0.00085 | 0.0574938 | 24.6566 | 8.80509 | -1.48556 | -2.11358 | KB871921.1:197020-204202 |
| XLOC_006633 | atp1a2a | 12.584 | 3.79992 | 5e-05 | 0.00738566 | 12.584 | 3.79992 | -1.72754 | -2.709 | KB871931.1:154312-213455 |
| XLOC_006714 | guca1c | 3.47989 | 10.9861 | 5e-05 | 0.00738566 | 3.47989 | 10.9861 | 1.65856 | 2.41026 | KB871938.1:1023233-1030654 |
| XLOC_006715 | thrsp | 138.457 | 14.6556 | 5e-05 | 0.00738566 | 138.457 | 14.6556 | -3.23991 | -3.87353 | KB871938.1:1035629-1036046 |
| XLOC_006754 | ENSAMXG00000010569 | 81.7739 | 16.2647 | 0.00045 | 0.0403574 | 81.7739 | 16.2647 | -2.3299 | -2.1627 | KB871938.1:4970804-5088665 |
| XLOC_006824 | lonrf1l | 2.60428 | 7.09716 | 0.00015 | 0.0178305 | 2.60428 | 7.09716 | 1.44636 | 2.25755 | KB871939.1:998737-1008850 |
| XLOC_006867 | slc44a5b | 3.0143 | 8.15188 | 7e-04 | 0.0518904 | 3.0143 | 8.15188 | 1.43531 | 1.99033 | KB871939.1:112534-168315 |
| XLOC_006902 | ENSAMXG00000014892 | 1.15661 | 3.16266 | 1e-04 | 0.0124776 | 1.15661 | 3.16266 | 1.45123 | 2.28706 | KB871939.1:2448361-2495470 |
| XLOC_007020 | ENSAMXG00000014157 | 8.75821 | 0 | 2e-04 | 0.0216011 | 8.75821 | 0 | -Inf | NA | KB871956.1:282358-292207 |
| XLOC_007022 | cers3a | 3.20073 | 8.61021 | 0.00025 | 0.0254937 | 3.20073 | 8.61021 | 1.42765 | 2.17536 | KB871956.1:344505-378639 |
| XLOC_007029 | ENSAMXG00000004919 | 2.34166 | 7.6518 | 0.00015 | 0.0178305 | 2.34166 | 7.6518 | 1.70827 | 2.26338 | KB871957.1:267374-285774 |
| XLOC_007097 | si:ch73-366l1.5 | 31.1437 | 8.53223 | 5e-05 | 0.00738566 | 31.1437 | 8.53223 | -1.86795 | -2.84312 | KB871968.1:178450-191470 |
| XLOC_007139 | ENSAMXG00000017732 | 18.9833 | 7.30933 | 7e-04 | 0.0518904 | 18.9833 | 7.30933 | -1.37692 | -2.02268 | KB871971.1:44926-183149 |
| XLOC_007244 | acsl3a | 1.30106 | 3.53938 | 0.00035 | 0.0338036 | 1.30106 | 3.53938 | 1.44381 | 2.01865 | KB871983.1:414440-478193 |
| XLOC_007276 | opn1sw2 | 18.629 | 50.3663 | 5e-05 | 0.00738566 | 18.629 | 50.3663 | 1.43491 | 2.31712 | KB871987.1:241053-248021 |
| XLOC_007454 | hjv | 6.36292 | 1.99583 | 5e-05 | 0.00738566 | 6.36292 | 1.99583 | -1.6727 | -2.47429 | KB872011.1:25030-30978 |
| XLOC_007827 | ENSAMXG00000016413 | 18.8718 | 4.65029 | 5e-05 | 0.00738566 | 18.8718 | 4.65029 | -2.02084 | -2.40919 | KB872051.1:117984-119391 |
| XLOC_007828 | ENSAMXG00000016426 | 13.2634 | 2.54315 | 5e-05 | 0.00738566 | 13.2634 | 2.54315 | -2.38277 | -2.58315 | KB872051.1:128145-129335 |
| XLOC_007831 | hbaa2 | 4.63625 | 14.6156 | 0.00105 | 0.0689328 | 4.63625 | 14.6156 | 1.65648 | 1.95051 | KB872051.1:192831-193838 |
| XLOC_007843 | ENSAMXG00000027508 | 15.1346 | 3.59669 | 5e-05 | 0.00738566 | 15.1346 | 3.59669 | -2.07311 | -2.57358 | KB872053.1:242757-247769 |
| XLOC_007871 | ENSAMXG00000014852 | 1.46566 | 4.56649 | 0.00115 | 0.0742841 | 1.46566 | 4.56649 | 1.63954 | 2.03722 | KB872056.1:223244-234328 |
| XLOC_007938 | lims1 | 11.6389 | 4.71026 | 0.00035 | 0.0338036 | 11.6389 | 4.71026 | -1.30508 | -1.98877 | KB872067.1:60303-75465 |
| XLOC_007949 | ENSAMXG00000011973 | 4.27761 | 0.542253 | 0.00175 | 0.0987521 | 4.27761 | 0.542253 | -2.97977 | -2.67761 | KB872068.1:62057-64354 |
| XLOC_008008 | ENSAMXG00000002825 | 84.4957 | 35.7196 | 0.00145 | 0.0861806 | 84.4957 | 35.7196 | -1.24216 | -1.93292 | KB872073.1:67872-84597 |
| XLOC_008156 | oit3 | 18.2981 | 8.02205 | 0.00155 | 0.0902549 | 18.2981 | 8.02205 | -1.18965 | -1.84166 | KB872081.1:3943983-3962613 |
| XLOC_008165 | ENSAMXG00000004102 | 36.2807 | 12.7707 | 4e-04 | 0.0372019 | 36.2807 | 12.7707 | -1.50636 | -2.08899 | KB872081.1:5025369-5034203 |
| XLOC_008312 | ENSAMXG00000005092 | 160.199 | 53.533 | 2e-04 | 0.0216011 | 160.199 | 53.533 | -1.58136 | -2.221 | KB872099.1:5050-16559 |
| XLOC_008418 | ENSAMXG00000001148 | 68.0436 | 15.6074 | 5e-05 | 0.00738566 | 68.0436 | 15.6074 | -2.12422 | -3.14949 | KB872110.1:134175-138156 |
| XLOC_008522 | fgfbp2a | 14.1128 | 32.843 | 0.00085 | 0.0574938 | 14.1128 | 32.843 | 1.21859 | 1.94405 | KB872123.1:87872-90425 |
| XLOC_008553 | arr3a | 13.9001 | 58.9019 | 5e-05 | 0.00738566 | 13.9001 | 58.9019 | 2.08322 | 3.07692 | KB872126.1:165767-172954 |
| XLOC_008650 | ENSAMXG00000010829 | 5.18706 | 1.51229 | 6e-04 | 0.0484072 | 5.18706 | 1.51229 | -1.77818 | -2.20244 | KB872142.1:228352-245718 |
| XLOC_008843 | apoa1b | 1185.73 | 290.254 | 8e-04 | 0.0556097 | 1185.73 | 290.254 | -2.0304 | -2.21997 | KB872172.1:161545-162549 |
| XLOC_009073 | slc34a2a | 4.59594 | 1.03457 | 5e-05 | 0.00738566 | 4.59594 | 1.03457 | -2.15133 | -2.72931 | KB872220.1:103546-112146 |
| XLOC_009276 | ENSAMXG00000002779 | 3.77377 | 13.5332 | 2e-04 | 0.0216011 | 3.77377 | 13.5332 | 1.84242 | 2.52683 | KB872260.1:223227-232193 |
| XLOC_009284 | ENSAMXG00000012870 | 6.91874 | 0 | 5e-05 | 0.00738566 | 6.91874 | 0 | -Inf | NA | KB872262.1:55697-59406 |
| XLOC_009374 | ENSAMXG00000004416 | 3.32707 | 0.709329 | 5e-05 | 0.00738566 | 3.32707 | 0.709329 | -2.22972 | -3.09356 | KB872286.1:83633-164522 |
| XLOC_009400 | ENSAMXG00000004721 | 16.0445 | 7.12806 | 0.00175 | 0.0987521 | 16.0445 | 7.12806 | -1.1705 | -1.76127 | KB872293.1:20-20286 |
| XLOC_009443 | abcb11b | 6.71758 | 0.910457 | 5e-05 | 0.00738566 | 6.71758 | 0.910457 | -2.88328 | -3.91516 | KB872295.1:2665897-2687825 |
| XLOC_009522 | ENSAMXG00000018710 | 47.8519 | 21.0951 | 0.00115 | 0.0742841 | 47.8519 | 21.0951 | -1.18167 | -1.89171 | KB872295.1:2651583-2658581 |
| XLOC_009582 | pyroxd2 | 12.6538 | 4.95315 | 0.00045 | 0.0403574 | 12.6538 | 4.95315 | -1.35315 | -2.07949 | KB872296.1:3963794-3980918 |
| XLOC_009613 | ENSAMXG00000001960 | 2.73837 | 7.05334 | 6e-04 | 0.0484072 | 2.73837 | 7.05334 | 1.36499 | 2.07965 | KB872296.1:2008216-2033838 |
| XLOC_009799 | noxo1a | 2.34339 | 7.36835 | 0.00025 | 0.0254937 | 2.34339 | 7.36835 | 1.65275 | 2.26632 | KB872330.1:2661-6570 |
| XLOC_009836 | zgc:194242 | 0.421738 | 6.8048 | 5e-05 | 0.00738566 | 0.421738 | 6.8048 | 4.01213 | 3.96289 | KB872337.1:1304-8307 |
| XLOC_009839 | tulp1a | 0.723851 | 5.85016 | 5e-05 | 0.00738566 | 0.723851 | 5.85016 | 3.01471 | 4.03184 | KB872337.1:114650-123319 |
| XLOC_010145 | ENSAMXG00000027789 | 0 | 1.80021 | 5e-05 | 0.00738566 | 0 | 1.80021 | Inf | NA | KB872413.1:25255-30416 |
| XLOC_010174 | ENSAMXG00000028677 | 13.4015 | 4.46936 | 6e-04 | 0.0484072 | 13.4015 | 4.46936 | -1.58426 | -2.12762 | KB872420.1:11056-13767 |
| XLOC_010316 | ENSAMXG00000025139 | 839.084 | 239.941 | 5e-05 | 0.00738566 | 839.084 | 239.941 | -1.80613 | -2.39668 | KB872462.1:39854-40028 |
| XLOC_010317 | ENSAMXG00000016276 | 29.6083 | 9.74245 | 1e-04 | 0.0124776 | 29.6083 | 9.74245 | -1.60365 | -2.29791 | KB872462.1:45007-46706 |
| XLOC_010374 | ENSAMXG00000014782 | 83.3677 | 23.6903 | 5e-05 | 0.00738566 | 83.3677 | 23.6903 | -1.81519 | -2.59629 | KB872486.1:82927-86298 |
| XLOC_010428 | ENSAMXG00000027402 | 1.55282 | 5.57506 | 2e-04 | 0.0216011 | 1.55282 | 5.57506 | 1.8441 | 2.50389 | KB872502.1:44788-47865 |
| XLOC_010600 | nfil3-6 | 4.46653 | 10.2847 | 0.0017 | 0.0964726 | 4.46653 | 10.2847 | 1.20328 | 1.80972 | KB872561.1:31281-32529 |
| XLOC_010648 | lad1 | 17.5937 | 39.3944 | 0.0017 | 0.0964726 | 17.5937 | 39.3944 | 1.16293 | 1.85973 | KB872583.1:105956-166287 |
| XLOC_010723 | ISYNA1 | 5.06362 | 12.7235 | 0.0017 | 0.0964726 | 5.06362 | 12.7235 | 1.32925 | 1.83118 | KB872617.1:42207-55231 |
| XLOC_010761 | ENSAMXG00000014761 | 5.0134 | 0.674813 | 5e-05 | 0.00738566 | 5.0134 | 0.674813 | -2.89323 | -2.86669 | KB872634.1:7919-17878 |
| XLOC_010795 | ENSAMXG00000025773 | 11.1455 | 1.18907 | 5e-05 | 0.00738566 | 11.1455 | 1.18907 | -3.22855 | -3.80008 | KB872649.1:63682-64978 |
| XLOC_010828 | ENSAMXG00000020730 | 91.073 | 27.146 | 2e-04 | 0.0216011 | 91.073 | 27.146 | -1.74628 | -2.10939 | KB872661.1:16537-18216 |
| XLOC_010858 | ENSAMXG00000000310 | 50.8205 | 21.3757 | 7e-04 | 0.0518904 | 50.8205 | 21.3757 | -1.24944 | -1.98511 | KB872672.1:53327-62597 |
| XLOC_011019 | ENSAMXG00000015089 | 9.65121 | 28.4598 | 1e-04 | 0.0124776 | 9.65121 | 28.4598 | 1.56014 | 2.44445 | KB872765.1:20868-31305 |
| XLOC_011042 | opn1mw1 | 5.61628 | 53.6355 | 5e-05 | 0.00738566 | 5.61628 | 53.6355 | 3.2555 | 4.76878 | KB872775.1:4150-8361 |
| XLOC_011065 | rorcb | 2.69209 | 6.71795 | 0.0013 | 0.0811047 | 2.69209 | 6.71795 | 1.3193 | 1.98158 | KB872789.1:22775-33957 |
| XLOC_011070 | ENSAMXG00000017636 | 131.845 | 53.3926 | 0.0014 | 0.0852261 | 131.845 | 53.3926 | -1.30413 | -1.8312 | KB872795.1:22420-23517 |
| XLOC_011143 | zgc:194930 | 4.4791 | 10.1122 | 0.00145 | 0.0861806 | 4.4791 | 10.1122 | 1.17481 | 1.87671 | KB872818.1:2447385-2478825 |
| XLOC_011258 | afp4 | 1693.33 | 542.51 | 5e-05 | 0.00738566 | 1693.33 | 542.51 | -1.64214 | -2.28476 | KB872819.1:3699762-3701692 |
| XLOC_011323 | ENSAMXG00000008228 | 2.83841 | 0.218586 | 1e-04 | 0.0124776 | 2.83841 | 0.218586 | -3.69881 | -3.65124 | KB872847.1:10343-13195 |
| XLOC_011332 | ENSAMXG00000000014 | 6.87697 | 19.705 | 0.00125 | 0.0789662 | 6.87697 | 19.705 | 1.51871 | 1.95895 | KB872850.1:43821-52867 |
| XLOC_011387 | ENSAMXG00000005597 | 79.0233 | 384.325 | 5e-05 | 0.00738566 | 79.0233 | 384.325 | 2.28198 | 2.69331 | KB872885.1:9359-15649 |
| XLOC_011414 | ENSAMXG00000002780 | 4.86323 | 1.36659 | 0.0017 | 0.0964726 | 4.86323 | 1.36659 | -1.83133 | -2.09229 | KB872914.1:11905-14091 |
| XLOC_011581 | serpina7 | 14.2071 | 4.32541 | 2e-04 | 0.0216011 | 14.2071 | 4.32541 | -1.71571 | -2.33065 | KB873067.1:15187-21113 |
| XLOC_011640 | ENSAMXG00000025083 | 51.7491 | 9.08725 | 4e-04 | 0.0372019 | 51.7491 | 9.08725 | -2.50962 | -2.58218 | KB873141.1:91-1784 |
| XLOC_011673 | ENSAMXG00000015333 | 1.00377 | 4.33012 | 0.001 | 0.0665199 | 1.00377 | 4.33012 | 2.10897 | 2.11181 | KB873175.1:243-4305 |
| XLOC_011884 | fgb | 90.2955 | 24.1439 | 5e-05 | 0.00738566 | 90.2955 | 24.1439 | -1.90299 | -2.43536 | KB873510.1:1452-18168 |
| XLOC_012011 | gatm | 187.446 | 53.6218 | 5e-05 | 0.00738566 | 187.446 | 53.6218 | -1.80558 | -2.63116 | KB873821.1:646-9057 |
| XLOC_012121 | ENSAMXG00000001171 | 5.87434 | 0.608114 | 5e-05 | 0.00738566 | 5.87434 | 0.608114 | -3.27201 | -3.72064 | KB874083.1:1207-5017 |
| XLOC_012207 | ENSAMXG00000028681 | 224.641 | 88.3755 | 7e-04 | 0.0518904 | 224.641 | 88.3755 | -1.3459 | -2.03825 | KB874410.1:10781-11970 |
| XLOC_012228 | ENSAMXG00000027658 | 1.88486 | 0 | 5e-05 | 0.00738566 | 1.88486 | 0 | -Inf | NA | KB874549.1:1179-4782 |
| XLOC_012243 | socs3a | 8.69069 | 23.5974 | 0.00035 | 0.0338036 | 8.69069 | 23.5974 | 1.44109 | 2.16173 | KB874601.1:1469-2093 |
| XLOC_012247 | ENSAMXG00000007322 | 0.846368 | 7.29867 | 1e-04 | 0.0124776 | 0.846368 | 7.29867 | 3.10828 | 3.24384 | KB874626.1:2926-4177 |
| XLOC_012491 | ENSAMXG00000004196,ENSAMXG00000027240 | 12.7805 | 269.836 | 5e-05 | 0.00738566 | 12.7805 | 269.836 | 4.40006 | 9.04013 | KB876205.1:905-9614 |
| XLOC_012502 | ENSAMXG00000019672 | 21.6104 | 8.87119 | 0.00165 | 0.0947053 | 21.6104 | 8.87119 | -1.28453 | -1.84507 | KB876219.1:1225-8155 |
| XLOC_012601 | dhrs13l1 | 17.3383 | 56.2398 | 5e-05 | 0.00738566 | 17.3383 | 56.2398 | 1.69763 | 2.4985 | KB876513.1:1771-6365 |
| XLOC_012720 | RF00004 | 289.548 | 0 | 0.00135 | 0.0831906 | 289.548 | 0 | -Inf | NA | KB876961.1:5667-5785 |
| XLOC_013073 | ENSAMXG00000015463 | 1.97724 | 22.9892 | 5e-05 | 0.00738566 | 1.97724 | 22.9892 | 3.5394 | 3.75024 | KB881556.1:252-1075 |
| XLOC_013084 | plpp7 | 14.3087 | 5.64829 | 0.00025 | 0.0254937 | 14.3087 | 5.64829 | -1.34101 | -2.12568 | KB882080.1:695248-703885 |
| XLOC_013156 | ENSAMXG00000027417 | 3.30734 | 0.937835 | 0.0012 | 0.0770185 | 3.30734 | 0.937835 | -1.81827 | -1.80971 | KB882080.1:4122615-4165750 |
| XLOC_013203 | si:dkey-88e18.2 | 1.38913 | 4.97042 | 8e-04 | 0.0556097 | 1.38913 | 4.97042 | 1.83918 | 1.99927 | KB882081.1:4308909-4313435 |
| XLOC_013234 | ENSAMXG00000003598 | 6.33514 | 1.44903 | 0.00035 | 0.0338036 | 6.33514 | 1.44903 | -2.12829 | -2.57682 | KB882081.1:3219975-3223639 |
| XLOC_013244 | tnni1c | 59.3591 | 24.9681 | 4e-04 | 0.0372019 | 59.3591 | 24.9681 | -1.24938 | -1.9611 | KB882081.1:4023977-4027183 |
| XLOC_013434 | obsl1a | 3.68128 | 1.59434 | 0.00065 | 0.0502225 | 3.68128 | 1.59434 | -1.20725 | -1.9129 | KB882082.1:3810391-3844914 |
| XLOC_013544 | ENSAMXG00000007167 | 14.0733 | 4.54104 | 0.00025 | 0.0254937 | 14.0733 | 4.54104 | -1.63187 | -2.21525 | KB882083.1:1898152-1908995 |
| XLOC_013638 | minpp1a | 6.28503 | 23.48 | 8e-04 | 0.0556097 | 6.28503 | 23.48 | 1.90144 | 1.96284 | KB882084.1:832325-842610 |
| XLOC_013678 | si:ch211-132f19.7 | 1.79756 | 0.474137 | 0.00025 | 0.0254937 | 1.79756 | 0.474137 | -1.92267 | -2.41254 | KB882084.1:4432035-4450238 |
| XLOC_013751 | ENSAMXG00000006428 | 1.49611 | 5.07163 | 0.00055 | 0.0450978 | 1.49611 | 5.07163 | 1.76123 | 2.08733 | KB882085.1:1710369-1713506 |
| XLOC_013762 | ENSAMXG00000006536 | 8.65892 | 29.9708 | 5e-05 | 0.00738566 | 8.65892 | 29.9708 | 1.7913 | 2.81043 | KB882085.1:2727659-2738109 |
| XLOC_013804 | f2 | 44.3624 | 15.4716 | 5e-04 | 0.0432953 | 44.3624 | 15.4716 | -1.51972 | -2.08741 | KB882086.1:3066893-3074937 |
| XLOC_013843 | ENSAMXG00000021040 | 3.00057 | 9.78594 | 0.00015 | 0.0178305 | 3.00057 | 9.78594 | 1.70548 | 2.32338 | KB882086.1:2962328-2965271 |
| XLOC_013915 | cacna2d1a | 9.12871 | 3.60254 | 0.00045 | 0.0403574 | 9.12871 | 3.60254 | -1.3414 | -2.04831 | KB882087.1:3261405-3323962 |
| XLOC_013950 | cldn18 | 14.3133 | 87.1471 | 5e-05 | 0.00738566 | 14.3133 | 87.1471 | 2.6061 | 2.96862 | KB882088.1:2214646-2223396 |
| XLOC_014159 | ENSAMXG00000010068 | 43.151 | 16.2052 | 0.00075 | 0.0540027 | 43.151 | 16.2052 | -1.41294 | -1.93706 | KB882090.1:1681088-1682458 |
| XLOC_014216 | ENSAMXG00000010163 | 0 | 2.3057 | 5e-05 | 0.00738566 | 0 | 2.3057 | Inf | NA | KB882090.1:1920322-1924962 |
| XLOC_014269 | tdo2a | 9.30809 | 2.90932 | 0.00025 | 0.0254937 | 9.30809 | 2.90932 | -1.6778 | -2.17427 | KB882091.1:489980-497676 |
| XLOC_014425 | fkbp9 | 25.2509 | 5.74246 | 5e-05 | 0.00738566 | 25.2509 | 5.74246 | -2.13659 | -3.32982 | KB882093.1:646064-654177 |
| XLOC_014455 | ENSAMXG00000028818 | 46.0104 | 6.83705 | 5e-05 | 0.00738566 | 46.0104 | 6.83705 | -2.75051 | -3.85869 | KB882094.1:71774-82002 |
| XLOC_014633 | grp | 1.91106 | 9.94569 | 5e-05 | 0.00738566 | 1.91106 | 9.94569 | 2.37969 | 2.92764 | KB882096.1:1162040-1168823 |
| XLOC_014802 | tmem82 | 5.41818 | 1.85308 | 0.00085 | 0.0574938 | 5.41818 | 1.85308 | -1.54788 | -2.01365 | KB882098.1:305735-310064 |
| XLOC_014878 | map1lc3cl | 6.03962 | 21.366 | 5e-05 | 0.00738566 | 6.03962 | 21.366 | 1.82278 | 2.54464 | KB882099.1:90771-93011 |
| XLOC_015020 | duox | 6.37988 | 36.0034 | 5e-05 | 0.00738566 | 6.37988 | 36.0034 | 2.49653 | 2.90409 | KB882100.1:2000619-2019230 |
| XLOC_015028 | asb15a | 3.41135 | 1.00845 | 0.00025 | 0.0254937 | 3.41135 | 1.00845 | -1.7582 | -2.3118 | KB882100.1:2710426-2716943 |
| XLOC_015067 | rab3da | 10.5606 | 23.8306 | 0.00135 | 0.0831906 | 10.5606 | 23.8306 | 1.17413 | 1.87603 | KB882101.1:1950042-1972519 |
| XLOC_015086 | ENSAMXG00000000481 | 7.68308 | 2.01204 | 3e-04 | 0.0296882 | 7.68308 | 2.01204 | -1.93302 | -2.26432 | KB882101.1:308357-310769 |
| XLOC_015239 | ENSAMXG00000005638 | 2.1106 | 0 | 5e-05 | 0.00738566 | 2.1106 | 0 | -Inf | NA | KB882103.1:688962-689632 |
| XLOC_015368 | rsph14 | 7.61391 | 1.25619 | 5e-05 | 0.00738566 | 7.61391 | 1.25619 | -2.59958 | -3.00133 | KB882105.1:515647-520846 |
| XLOC_015415 | si:busm1-266f07.2 | 55.2484 | 5.36906 | 5e-05 | 0.00738566 | 55.2484 | 5.36906 | -3.36319 | -4.58697 | KB882105.1:212135-221320 |
| XLOC_015421 | star | 1.22599 | 4.77582 | 5e-05 | 0.00738566 | 1.22599 | 4.77582 | 1.9618 | 2.448 | KB882105.1:400138-404684 |
| XLOC_015427 | si:ch211-170d8.5 | 3.81639 | 0.930637 | 2e-04 | 0.0216011 | 3.81639 | 0.930637 | -2.03592 | -2.4455 | KB882105.1:1559551-1568711 |
| XLOC_015638 | zgc:162144 | 3.89968 | 1.25653 | 8e-04 | 0.0556097 | 3.89968 | 1.25653 | -1.63392 | -2.06812 | KB882108.1:306368-308320 |
| XLOC_015734 | slc8a3 | 4.78051 | 1.85809 | 0.0014 | 0.0852261 | 4.78051 | 1.85809 | -1.36335 | -1.91299 | KB882109.1:2933085-3008501 |
| XLOC_015751 | ENSAMXG00000011560 | 9.54339 | 42.8343 | 1e-04 | 0.0124776 | 9.54339 | 42.8343 | 2.16619 | 2.53697 | KB882110.1:206963-208029 |
| XLOC_015769 | ENSAMXG00000028550 | 24.6941 | 134.174 | 3e-04 | 0.0296882 | 24.6941 | 134.174 | 2.44187 | 4.5531 | KB882110.1:2696591-2697884 |
| XLOC_015813 | ENSAMXG00000007189 | 2114.1 | 7716.11 | 7e-04 | 0.0518904 | 2114.1 | 7716.11 | 1.86783 | 1.98399 | KB882111.1:1213159-1218786 |
| XLOC_016155 | calcoco1b | 0.168095 | 1.89092 | 5e-05 | 0.00738566 | 0.168095 | 1.89092 | 3.49174 | 3.55109 | KB882116.1:1649691-1665179 |
| XLOC_016157 | nr1d4b | 9.43547 | 3.81497 | 0.00065 | 0.0502225 | 9.43547 | 3.81497 | -1.30642 | -2.02862 | KB882116.1:1829292-1850489 |
| XLOC_016289 | nkx6.2 | 2.00559 | 9.50662 | 5e-05 | 0.00738566 | 2.00559 | 9.50662 | 2.2449 | 2.95916 | KB882118.1:27043-29344 |
| XLOC_016404 | cabp5a | 11.0573 | 25.4383 | 0.00115 | 0.0742841 | 11.0573 | 25.4383 | 1.202 | 1.89508 | KB882119.1:203599-209419 |
| XLOC_016478 | nox1 | 7.13281 | 21.4823 | 5e-05 | 0.00738566 | 7.13281 | 21.4823 | 1.5906 | 2.3422 | KB882119.1:2377781-2392325 |
| XLOC_016636 | zgc:112408 | 3.10042 | 14.8977 | 1e-04 | 0.0124776 | 3.10042 | 14.8977 | 2.26455 | 2.70322 | KB882121.1:1709196-1714770 |
| XLOC_016812 | ENSAMXG00000025425 | 6.69814 | 33.1008 | 0.00125 | 0.0789662 | 6.69814 | 33.1008 | 2.30503 | 2.46293 | KB882124.1:1942746-1943496 |
| XLOC_016815 | rida | 34.3824 | 13.6325 | 4e-04 | 0.0372019 | 34.3824 | 13.6325 | -1.33462 | -2.034 | KB882124.1:2108955-2115104 |
| XLOC_016913 | prom2 | 11.7115 | 26.8014 | 0.00145 | 0.0861806 | 11.7115 | 26.8014 | 1.19438 | 1.90546 | KB882125.1:851909-874460 |
| XLOC_016970 | ENSAMXG00000012464 | 5.5153 | 1.34483 | 5e-05 | 0.00738566 | 5.5153 | 1.34483 | -2.03601 | -2.62552 | KB882125.1:2097923-2107121 |
| XLOC_016987 | si:dkey-61p9.11 | 4.52351 | 1.33199 | 1e-04 | 0.0124776 | 4.52351 | 1.33199 | -1.76386 | -2.39382 | KB882126.1:318388-326998 |
| XLOC_017269 | rtn2b | 21.6673 | 8.93054 | 0.00065 | 0.0502225 | 21.6673 | 8.93054 | -1.2787 | -1.98303 | KB882129.1:352176-365346 |
| XLOC_017677 | CRACR2A | 1.12663 | 3.77506 | 0.00055 | 0.0450978 | 1.12663 | 3.77506 | 1.74449 | 2.28683 | KB882135.1:97375-125255 |
| XLOC_017764 | slc1a7a | 2.09096 | 0.527558 | 2e-04 | 0.0216011 | 2.09096 | 0.527558 | -1.98676 | -2.39707 | KB882137.1:887543-921811 |
| XLOC_017952 | ENSAMXG00000018683 | 7.53352 | 2.57226 | 2e-04 | 0.0216011 | 7.53352 | 2.57226 | -1.55029 | -2.2039 | KB882139.1:2130673-2153246 |
| XLOC_017953 | ceacam1 | 8.10879 | 42.4682 | 5e-05 | 0.00738566 | 8.10879 | 42.4682 | 2.38883 | 2.99767 | KB882139.1:2170775-2187055 |
| XLOC_017991 | cnfn | 58.4859 | 236.735 | 0.00015 | 0.0178305 | 58.4859 | 236.735 | 2.01711 | 2.33068 | KB882139.1:2229954-2231596 |
| XLOC_018093 | rcn3 | 13.974 | 3.02097 | 5e-05 | 0.00738566 | 13.974 | 3.02097 | -2.20966 | -3.20665 | KB882142.1:1293779-1298434 |
| XLOC_018150 | ENSAMXG00000010386 | 1.1248 | 0 | 5e-05 | 0.00738566 | 1.1248 | 0 | -Inf | NA | KB882142.1:1634996-1656339 |
| XLOC_018187 | noxo1b | 6.97124 | 70.0027 | 5e-05 | 0.00738566 | 6.97124 | 70.0027 | 3.32792 | 4.0743 | KB882143.1:1198526-1206548 |
| XLOC_018209 | tefa | 14.5185 | 32.6502 | 7e-04 | 0.0518904 | 14.5185 | 32.6502 | 1.1692 | 1.91382 | KB882143.1:2307401-2311742 |
| XLOC_018289 | lrrc58a | 1.30221 | 3.77552 | 0.00135 | 0.0831906 | 1.30221 | 3.77552 | 1.53571 | 1.89232 | KB882144.1:970506-980419 |
| XLOC_018397 | p3h1 | 4.94728 | 2.12861 | 0.001 | 0.0665199 | 4.94728 | 2.12861 | -1.21672 | -1.84707 | KB882145.1:2066286-2082635 |
| XLOC_018467 | esrrd | 4.05731 | 12.2426 | 5e-05 | 0.00738566 | 4.05731 | 12.2426 | 1.59332 | 2.46542 | KB882146.1:1513311-1526512 |
| XLOC_018486 | ENSAMXG00000020645 | 393.498 | 141.315 | 5e-04 | 0.0432953 | 393.498 | 141.315 | -1.47744 | -2.18495 | KB882146.1:1286623-1287664 |
| XLOC_018494 | foxa3 | 6.76531 | 15.3178 | 0.0013 | 0.0811047 | 6.76531 | 15.3178 | 1.17898 | 1.87088 | KB882146.1:1859363-1865147 |
| XLOC_018523 | rgcc | 33.705 | 98.6289 | 5e-05 | 0.00738566 | 33.705 | 98.6289 | 1.54905 | 2.50061 | KB882147.1:1553242-1559475 |
| XLOC_018615 | ENSAMXG00000020762 | 2.54283 | 0.711618 | 0.00145 | 0.0861806 | 2.54283 | 0.711618 | -1.83726 | -2.05134 | KB882149.1:354205-419865 |
| XLOC_018701 | clic5a | 4.24356 | 1.59612 | 7e-04 | 0.0518904 | 4.24356 | 1.59612 | -1.4107 | -1.9045 | KB882150.1:1879199-1890582 |
| XLOC_018792 | ENSAMXG00000009725 | 18.941 | 51.8438 | 3e-04 | 0.0296882 | 18.941 | 51.8438 | 1.45266 | 2.07903 | KB882151.1:2828307-2836759 |
| XLOC_018793 | APOH | 8.92664 | 3.40406 | 0.0015 | 0.0878528 | 8.92664 | 3.40406 | -1.39086 | -1.83549 | KB882152.1:451566-468120 |
| XLOC_018827 | aanat1 | 27.3703 | 4.58755 | 5e-05 | 0.00738566 | 27.3703 | 4.58755 | -2.57682 | -3.37155 | KB882152.1:1429795-1431775 |
| XLOC_018850 | rsad2 | 2.33004 | 10.4055 | 5e-05 | 0.00738566 | 2.33004 | 10.4055 | 2.15892 | 2.77737 | KB882153.1:1732446-1736226 |
| XLOC_019129 | fetub | 19.1377 | 6.40619 | 1e-04 | 0.0124776 | 19.1377 | 6.40619 | -1.57888 | -2.23955 | KB882159.1:316425-328721 |
| XLOC_019196 | tm6sf2 | 8.66805 | 3.00263 | 1e-04 | 0.0124776 | 8.66805 | 3.00263 | -1.52948 | -2.34197 | KB882160.1:2065541-2087059 |
| XLOC_019233 | slc5a8 | 0.618203 | 2.59508 | 0.00065 | 0.0502225 | 0.618203 | 2.59508 | 2.06962 | 2.11096 | KB882162.1:1869266-1878015 |
| XLOC_019321 | tnni4b.2 | 72.4225 | 28.0282 | 0.00065 | 0.0502225 | 72.4225 | 28.0282 | -1.36956 | -2.06959 | KB882164.1:69935-72299 |
| XLOC_019391 | ENSAMXG00000009469 | 305.971 | 89.4519 | 7e-04 | 0.0518904 | 305.971 | 89.4519 | -1.77421 | -2.1061 | KB882165.1:1308251-1310558 |
| XLOC_019393 | ENSAMXG00000009481 | 24.4729 | 6.44703 | 0.00065 | 0.0502225 | 24.4729 | 6.44703 | -1.92448 | -2.03948 | KB882165.1:1330064-1348296 |
| XLOC_019441 | me1 | 68.1913 | 18.7993 | 5e-05 | 0.00738566 | 68.1913 | 18.7993 | -1.85891 | -2.75406 | KB882166.1:2099378-2196189 |
| XLOC_019461 | ENSAMXG00000008750 | 2.75925 | 14.4991 | 5e-05 | 0.00738566 | 2.75925 | 14.4991 | 2.39361 | 3.82746 | KB882166.1:1824805-1848177 |
| XLOC_019467 | ENSAMXG00000008922 | 1.64697 | 5.64861 | 3e-04 | 0.0296882 | 1.64697 | 5.64861 | 1.77809 | 2.41595 | KB882166.1:2531268-2533496 |
| XLOC_019560 | si:ch211-63p21.8 | 0.384448 | 1.54061 | 0.00015 | 0.0178305 | 0.384448 | 1.54061 | 2.00264 | 2.54532 | KB882169.1:123635-128754 |
| XLOC_019685 | igfn1.3 | 5.57065 | 2.14095 | 0.00015 | 0.0178305 | 5.57065 | 2.14095 | -1.37959 | -2.13406 | KB882171.1:4941586-4966155 |
| XLOC_019690 | ptgis | 0.797064 | 2.39421 | 0.00075 | 0.0540027 | 0.797064 | 2.39421 | 1.58678 | 1.95254 | KB882171.1:5206751-5226126 |
| XLOC_019772 | rbp7b | 37.7257 | 9.62923 | 5e-05 | 0.00738566 | 37.7257 | 9.62923 | -1.97005 | -3.11842 | KB882171.1:5730311-5734124 |
| XLOC_019868 | vill | 5.25876 | 22.0608 | 5e-05 | 0.00738566 | 5.25876 | 22.0608 | 2.06869 | 2.66002 | KB882174.1:2304249-2332392 |
| XLOC_019943 | CLDN18 | 26.2224 | 183.058 | 1e-04 | 0.0124776 | 26.2224 | 183.058 | 2.80343 | 2.94561 | KB882176.1:135549-140398 |
| XLOC_019971 | ENSAMXG00000005446 | 8.47209 | 3.49831 | 8e-04 | 0.0556097 | 8.47209 | 3.49831 | -1.27606 | -1.95463 | KB882177.1:430529-441690 |
| XLOC_019973 | ENSAMXG00000028341 | 3.07469 | 1.03083 | 0.0015 | 0.0878528 | 3.07469 | 1.03083 | -1.57663 | -1.90166 | KB882177.1:508417-518105 |
| XLOC_019983 | frem3 | 6.19939 | 2.87584 | 0.00125 | 0.0789662 | 6.19939 | 2.87584 | -1.10814 | -1.81975 | KB882177.1:2087756-2162568 |
| XLOC_020107 | capn3b | 4.18702 | 1.09168 | 5e-05 | 0.00738566 | 4.18702 | 1.09168 | -1.93938 | -2.69238 | KB882181.1:493429-517067 |
| XLOC_020174 | zmp:0000000846 | 5.76813 | 28.0792 | 5e-05 | 0.00738566 | 5.76813 | 28.0792 | 2.28333 | 3.10672 | KB882182.1:289544-377076 |
| XLOC_020324 | si:dkey-183i3.5 | 116.963 | 44.9713 | 5e-04 | 0.0432953 | 116.963 | 44.9713 | -1.37898 | -1.98152 | KB882184.1:1833668-1842014 |
| XLOC_020338 | ghrl | 8.32656 | 46.2696 | 5e-05 | 0.00738566 | 8.32656 | 46.2696 | 2.47427 | 3.16788 | KB882185.1:955034-956773 |
| XLOC_020358 | fkbp5 | 3.16764 | 12.8911 | 5e-05 | 0.00738566 | 3.16764 | 12.8911 | 2.02489 | 2.87151 | KB882185.1:1474306-1492367 |
| XLOC_020421 | f9a | 1.79665 | 0.510339 | 5e-04 | 0.0432953 | 1.79665 | 0.510339 | -1.81578 | -2.18805 | KB882186.1:298362-306971 |
| XLOC_020515 | trhra | 0.557703 | 1.51277 | 0.0016 | 0.0923632 | 0.557703 | 1.51277 | 1.43963 | 1.88077 | KB882188.1:127702-138105 |
| XLOC_020582 | zgc:172270 | 10.5903 | 4.37526 | 0.0015 | 0.0878528 | 10.5903 | 4.37526 | -1.27531 | -1.81519 | KB882189.1:407700-411259 |
| XLOC_020691 | RF00411 | 0 | 36.4129 | 0.00145 | 0.0861806 | 0 | 36.4129 | Inf | NA | KB882192.1:1879264-1879400 |
| XLOC_020745 | ptgdsb.1 | 46.8369 | 186.845 | 5e-05 | 0.00738566 | 46.8369 | 186.845 | 1.99612 | 3.19167 | KB882193.1:1400216-1402995 |
| XLOC_020783 | f10 | 10.4939 | 3.58352 | 0.00045 | 0.0403574 | 10.4939 | 3.58352 | -1.5501 | -2.12136 | KB882194.1:1862976-1873343 |
| XLOC_020842 | ENSAMXG00000020478 | 1.33991 | 5.45259 | 4e-04 | 0.0372019 | 1.33991 | 5.45259 | 2.02481 | 2.29157 | KB882196.1:160868-173864 |
| XLOC_021022 | myoz2a | 32.0886 | 9.48206 | 5e-05 | 0.00738566 | 32.0886 | 9.48206 | -1.75879 | -2.52943 | KB882199.1:158356-174366 |
| XLOC_021193 | ENSAMXG00000028390 | 25.5123 | 8.99533 | 0.00055 | 0.0450978 | 25.5123 | 8.99533 | -1.50395 | -2.01482 | KB882202.1:2073617-2084028 |
| XLOC_021216 | si:zfos-223e1.2 | 6.32949 | 0.938575 | 5e-05 | 0.00738566 | 6.32949 | 0.938575 | -2.75355 | -3.89346 | KB882202.1:3836973-3885323 |
| XLOC_021299 | vtcn1 | 1.57467 | 12.2831 | 5e-05 | 0.00738566 | 1.57467 | 12.2831 | 2.96355 | 3.30079 | KB882203.1:2048678-2052087 |
| XLOC_021346 | EIF5AL1 | 64.8907 | 27.7358 | 0.0014 | 0.0852261 | 64.8907 | 27.7358 | -1.22626 | -1.90473 | KB882205.1:761165-766595 |
| XLOC_021415 | hpda | 33.1709 | 10.1052 | 2e-04 | 0.0216011 | 33.1709 | 10.1052 | -1.71482 | -2.3654 | KB882207.1:1071964-1110817 |
| XLOC_021445 | hspb1 | 58.7202 | 25.5665 | 7e-04 | 0.0518904 | 58.7202 | 25.5665 | -1.1996 | -1.93777 | KB882207.1:1632877-1642891 |
| XLOC_021617 | agt | 5.39916 | 1.87453 | 0.00085 | 0.0574938 | 5.39916 | 1.87453 | -1.52621 | -2.00331 | KB882210.1:608040-616510 |
| XLOC_021679 | gls2a | 6.02413 | 13.7149 | 0.0011 | 0.0719801 | 6.02413 | 13.7149 | 1.18692 | 1.83241 | KB882211.1:1237783-1270711 |
| XLOC_021786 | slc16a7 | 2.23961 | 0.429028 | 0.00055 | 0.0450978 | 2.23961 | 0.429028 | -2.3841 | -2.49175 | KB882214.1:536854-545219 |
| XLOC_021793 | si:ch73-269m23.5 | 5.17944 | 1.71751 | 0.00055 | 0.0450978 | 5.17944 | 1.71751 | -1.59248 | -2.13826 | KB882214.1:9545-14471 |
| XLOC_021804 | krt5 | 557.563 | 196.279 | 0.00075 | 0.0540027 | 557.563 | 196.279 | -1.50623 | -1.92066 | KB882214.1:724231-728166 |
| XLOC_021915 | dbpb | 4.68001 | 0.931464 | 5e-05 | 0.00738566 | 4.68001 | 0.931464 | -2.32894 | -3.0408 | KB882219.1:812678-823637 |
| XLOC_021990 | tdh | 28.7533 | 74.4462 | 0.00075 | 0.0540027 | 28.7533 | 74.4462 | 1.37247 | 2.07401 | KB882221.1:418728-427626 |
| XLOC_022058 | cry1bb | 7.1691 | 16.9325 | 4e-04 | 0.0372019 | 7.1691 | 16.9325 | 1.23993 | 2.01126 | KB882222.1:1131911-1144602 |
| XLOC_022096 | apobec2a | 74.6589 | 25.1353 | 5e-05 | 0.00738566 | 74.6589 | 25.1353 | -1.5706 | -2.45778 | KB882222.1:927578-928665 |
| XLOC_022122 | kng1 | 33.3538 | 12.3665 | 0.00105 | 0.0689328 | 33.3538 | 12.3665 | -1.43141 | -1.84292 | KB882223.1:700652-707023 |
| XLOC_022134 | ENSAMXG00000001567 | 12.9239 | 3.85645 | 5e-05 | 0.00738566 | 12.9239 | 3.85645 | -1.74469 | -2.42787 | KB882223.1:693259-698500 |
| XLOC_022209 | ENSAMXG00000011255 | 3.46259 | 0.791043 | 1e-04 | 0.0124776 | 3.46259 | 0.791043 | -2.13002 | -2.38538 | KB882226.1:586876-592871 |
| XLOC_022287 | ENSAMXG00000012686 | 21.9871 | 5.11093 | 5e-05 | 0.00738566 | 21.9871 | 5.11093 | -2.105 | -2.83134 | KB882227.1:978239-982001 |
| XLOC_022304 | ttr | 23.5395 | 9.2831 | 0.00055 | 0.0450978 | 23.5395 | 9.2831 | -1.3424 | -1.91798 | KB882228.1:265590-269296 |
| XLOC_022388 | nfil3-5 | 12.51 | 42.5922 | 5e-05 | 0.00738566 | 12.51 | 42.5922 | 1.76751 | 2.88125 | KB882230.1:1520805-1522581 |
| XLOC_022467 | dhrs7cb | 36.8643 | 12.793 | 5e-05 | 0.00738566 | 36.8643 | 12.793 | -1.52687 | -2.43824 | KB882231.1:1041292-1055122 |
| XLOC_022521 | ENSAMXG00000014315 | 7.85082 | 1.96306 | 5e-05 | 0.00738566 | 7.85082 | 1.96306 | -1.99974 | -2.65794 | KB882233.1:3476411-3489654 |
| XLOC_022604 | PLCB | 1.16374 | 3.26331 | 5e-05 | 0.00738566 | 1.16374 | 3.26331 | 1.48757 | 2.18254 | KB882233.1:6157952-6212655 |
| XLOC_022632 | cbln17 | 25.974 | 6.97346 | 0.00065 | 0.0502225 | 25.974 | 6.97346 | -1.89712 | -2.14317 | KB882234.1:1382594-1388624 |
| XLOC_022748 | IMPG2 | 4.8247 | 1.50829 | 1e-04 | 0.0124776 | 4.8247 | 1.50829 | -1.67753 | -2.36292 | KB882237.1:1046210-1099373 |
| XLOC_022789 | anxa6 | 59.7876 | 25.8033 | 8e-04 | 0.0556097 | 59.7876 | 25.8033 | -1.21229 | -1.86622 | KB882239.1:846264-881430 |
| XLOC_022881 | tfa | 211.861 | 60.2335 | 0.00045 | 0.0403574 | 211.861 | 60.2335 | -1.81448 | -2.16776 | KB882241.1:1709775-1723302 |
| XLOC_023022 | casq1a | 25.6961 | 7.42792 | 5e-05 | 0.00738566 | 25.6961 | 7.42792 | -1.79052 | -2.63776 | KB882246.1:808530-816311 |
| XLOC_023119 | fah | 26.7036 | 12.4046 | 0.00165 | 0.0947053 | 26.7036 | 12.4046 | -1.10616 | -1.81082 | KB882249.1:1683760-1717112 |
| XLOC_023153 | asb2a.1 | 0.513023 | 2.7796 | 0.00025 | 0.0254937 | 0.513023 | 2.7796 | 2.43778 | 2.67106 | KB882250.1:1110546-1115791 |
| XLOC_023194 | si:ch211-232m10.6 | 0.842842 | 2.38 | 0.00065 | 0.0502225 | 0.842842 | 2.38 | 1.49763 | 2.01653 | KB882251.1:861514-871137 |
| XLOC_023206 | ENSAMXG00000009598 | 234.387 | 59.699 | 5e-05 | 0.00738566 | 234.387 | 59.699 | -1.97311 | -2.71601 | KB882251.1:1492607-1500271 |
| XLOC_023301 | ARL14 | 4.83578 | 26.1987 | 0.00065 | 0.0502225 | 4.83578 | 26.1987 | 2.43767 | 2.48397 | KB882254.1:1191647-1192262 |
| XLOC_023352 | trim63a | 18.3331 | 2.05562 | 5e-05 | 0.00738566 | 18.3331 | 2.05562 | -3.1568 | -4.49833 | KB882256.1:514228-515290 |
| XLOC_023415 | ENSAMXG00000028514 | 1.80512 | 0 | 0.00035 | 0.0338036 | 1.80512 | 0 | -Inf | NA | KB882257.1:897719-899753 |
| XLOC_023576 | camk1gb | 7.04359 | 21.5279 | 1e-04 | 0.0124776 | 7.04359 | 21.5279 | 1.61183 | 2.49612 | KB882262.1:585977-605973 |
| XLOC_023658 | ENSAMXG00000025008 | 11.4043 | 41.6757 | 5e-05 | 0.00738566 | 11.4043 | 41.6757 | 1.86962 | 2.5223 | KB882265.1:1479264-1479678 |
| XLOC_023659 | ENSAMXG00000020401 | 13.8796 | 1.62433 | 0.00055 | 0.0450978 | 13.8796 | 1.62433 | -3.09505 | -3.14221 | KB882265.1:1502164-1502865 |
| XLOC_023687 | fbxo21 | 5.25162 | 14.739 | 5e-05 | 0.00738566 | 5.25162 | 14.739 | 1.4888 | 2.29414 | KB882266.1:1730495-1770191 |
| XLOC_023762 | si:ch1073-440b2.1 | 10.4369 | 25.4015 | 0.00045 | 0.0403574 | 10.4369 | 25.4015 | 1.28322 | 2.07947 | KB882269.1:246386-274675 |
| XLOC_023791 | si:dkey-220f10.4 | 3.82907 | 1.2969 | 0.00055 | 0.0450978 | 3.82907 | 1.2969 | -1.56192 | -2.05953 | KB882270.1:302818-310003 |
| XLOC_023852 | zgc:158404 | 13.399 | 4.48923 | 5e-05 | 0.00738566 | 13.399 | 4.48923 | -1.57758 | -2.36692 | KB882270.1:4030758-4041129 |
| XLOC_023950 | enpp7.2 | 5.34996 | 16.4123 | 5e-05 | 0.00738566 | 5.34996 | 16.4123 | 1.61718 | 2.38606 | KB882270.1:5657277-5668243 |
| XLOC_023962 | rs1a | 17.4585 | 43.4856 | 0.00015 | 0.0178305 | 17.4585 | 43.4856 | 1.31661 | 2.12548 | KB882271.1:45462-56630 |
| XLOC_023975 | si:dkey-18a10.3 | 7.40938 | 16.9341 | 0.00095 | 0.0638279 | 7.40938 | 16.9341 | 1.19251 | 1.84885 | KB882272.1:193251-203983 |
| XLOC_024005 | myl10 | 331.775 | 112.53 | 0.00135 | 0.0831906 | 331.775 | 112.53 | -1.5599 | -2.01332 | KB882272.1:489176-491899 |
| XLOC_024058 | si:dkey-183n20.15 | 9.33295 | 3.21923 | 1e-04 | 0.0124776 | 9.33295 | 3.21923 | -1.53562 | -2.03294 | KB882273.1:978216-986042 |
| XLOC_024060 | cyp2ad6 | 2.53474 | 7.96732 | 2e-04 | 0.0216011 | 2.53474 | 7.96732 | 1.65226 | 2.20046 | KB882273.1:1043530-1050357 |
| XLOC_024365 | abcb11a | 5.76304 | 0.680922 | 5e-05 | 0.00738566 | 5.76304 | 0.680922 | -3.08127 | -4.13732 | KB882284.1:888672-900259 |
| XLOC_024373 | ENSAMXG00000006171 | 10.6621 | 25.7904 | 8e-04 | 0.0556097 | 10.6621 | 25.7904 | 1.27435 | 1.95357 | KB882284.1:1287431-1292320 |
| XLOC_024375 | ENSAMXG00000005569 | 9.14128 | 34.7095 | 5e-05 | 0.00738566 | 9.14128 | 34.7095 | 1.92486 | 2.94981 | KB882284.1:390628-401042 |
| XLOC_024379 | TUBA4A | 99.7674 | 15.5236 | 5e-05 | 0.00738566 | 99.7674 | 15.5236 | -2.6841 | -4.09711 | KB882284.1:504439-507144 |
| XLOC_024386 | klhl41a | 8.44525 | 2.87167 | 0.00065 | 0.0502225 | 8.44525 | 2.87167 | -1.55625 | -2.1465 | KB882284.1:741078-746501 |
| XLOC_024388 | col28a2a | 2.70993 | 0.611707 | 5e-05 | 0.00738566 | 2.70993 | 0.611707 | -2.14735 | -3.05323 | KB882284.1:754399-776147 |
| XLOC_024488 | trpa1b | 0.608163 | 2.41025 | 1e-04 | 0.0124776 | 0.608163 | 2.41025 | 1.98665 | 2.5676 | KB882288.1:781656-839392 |
| XLOC_024551 | rgs9bp | 1.09637 | 3.81565 | 5e-05 | 0.00738566 | 1.09637 | 3.81565 | 1.79919 | 2.49079 | KB882290.1:1513855-1522129 |
| XLOC_024582 | UBAP1L | 3.72568 | 0.708624 | 5e-05 | 0.00738566 | 3.72568 | 0.708624 | -2.39441 | -3.11869 | KB882292.1:694761-714495 |
| XLOC_024590 | ENSAMXG00000003434 | 18.931 | 52.7914 | 8e-04 | 0.0556097 | 18.931 | 52.7914 | 1.47956 | 2.04781 | KB882292.1:1414437-1474066 |
| XLOC_024818 | VSIG2 | 2.34237 | 12.6587 | 7e-04 | 0.0518904 | 2.34237 | 12.6587 | 2.43409 | 2.46938 | KB882299.1:1085728-1098687 |
| XLOC_025145 | rnasel3 | 73.7627 | 22.5763 | 1e-04 | 0.0124776 | 73.7627 | 22.5763 | -1.70808 | -2.54725 | KB882311.1:1429665-1430118 |
| XLOC_025152 | ENSAMXG00000001701 | 145.931 | 51.2912 | 5e-05 | 0.00738566 | 145.931 | 51.2912 | -1.5085 | -2.33554 | KB882311.1:471525-477166 |
| XLOC_025153 | ENSAMXG00000001722 | 29.1152 | 217.014 | 5e-05 | 0.00738566 | 29.1152 | 217.014 | 2.89795 | 4.3711 | KB882311.1:481635-486402 |
| XLOC_025154 | apoa1a | 208.682 | 54.5999 | 5e-05 | 0.00738566 | 208.682 | 54.5999 | -1.93434 | -2.89141 | KB882311.1:585779-587980 |
| XLOC_025169 | SCTR | 1.89309 | 8.1624 | 5e-05 | 0.00738566 | 1.89309 | 8.1624 | 2.10825 | 3.05091 | KB882312.1:152821-170018 |
