# Supplementary material for: Phenotypic plasticity as a mechanism of cave colonization and adaptation
Source: eLife. 2020 Apr 21;9:e51830. doi: 10.7554/eLife.51830 (PMC7173965; doi:10.7554/eLife.51830)
Supplement: Supplementary file 1. [file elife-51830-supp1.docx]

Supplementary File 1. List of genes and primers used in RT-PCR experiments.

| Gene | Ensembl accession # | Primer A | Primer B |
| --- | --- | --- | --- |
| hsp90aa1.2 | ENSAMXG00000020572 | GCCAGACAATGGTGAGTCTATC | GATGCGCTTCTCCTCTGTATATT |
| tdo2a | ENSAMXG00000032882 | CCACCGGATCGTGATGATATT | AGGAGAGAGATACTCCCTGAAG |
| tph1a | ENSAMXG00000018104 | CTTGCCACTTGTTACTTCTTCAC | CAGTTCACTGATGGAGGACAG |
| aanat1 | ENSAMXG00000015728 | CCTTCATCATTGGCTCCCTAT | CACATCAGGATGGAGCCTTT |
| sst1.2 (1 of many) | ENSAMXG00000006540 | TGCACAGGAATGGAAGAAGAG | CCACTCGGACATCCTGTTTAG |
| inhbb | ENSAMXG00000003122 | CGGAGACAGATGACTCAACATT | CCAGGCAACAGCTTGAAGTA |
| rs1a | ENSAMXG00000033915 | CAGGAGGGAGTAGAGGACAAT | CCAACTAGAACTAGCGGTGATG |
| hpda | ENSAMXG00000015913 | CTGAAGACCCTGCAGGAATTA | CTCTGGATGACCTCTAGGAAGA |
| cry3b | ENSAMXG00000008895 | ATTTCGAGAGGCCCAAGATG | TGCTTCACCCGTTTGTAGAG |
| pdx1 | ENSAMXG00000031179 | GATCTCTGTCCAGAGCGAAAC | CGCTTGTTGTCCTCCACTT |
| per2 | ENSAMXG00000001431 | CGTCATCCAGCGAGAGTAAC | TCCTGAAAGGTTCTAGGTTTGG |
| ghrl | ENSAMXG00000002173 | TTACTTGTGGCTCCAGCTTC | AAGGTGCGCTCATCATTAGG |
| nr1d1 | ENSAMXG00000015855 | GGAACCTTTGAGGTGCTGAT | GTAGGTGGTGCCAGAGATAAAC |
| deptor | ENSAMXG00000042482 | GCTGTTGGAATGAAGGTGTG | TGAGGATGAGGTGACTGACT |
| duox | ENSAMXG00000000686 | ACGTCTTTGCTACGCACTAC | GGATCACCGTCAAGGTGTAAA |
| fads2 | ENSAMXG00000015974 | CATGCTGAAGATCCTGGTACTG | GCAGAGGAGGACCAATGAAA |
| dnmt3bb.1 | ENSAMXG00000019567 | TCAGCCAATGCTGTCATACG | TGGGCATTCAAACTCCTGTC |
| dnmt1 | ENSAMXG00000012182 | CGTCCGGAACTTTGTGTCTTT | ACTGTCCAGCCTGAAGTACA |
| tulp1a | ENSAMXG00000005179 | GTTCACAGCAAAGACAGTGATTAT | CGCACAGCGGGTACTTATAG |
| mob4 | ENSAMXG00000036359 | TCTGGCAGTGCAACAGTATATC | CGTACTTCCACACTCCTTCATC |
| tbcb | ENSAMXG00000038054 | TTGTTCAGCACCTCGGATAAG | CGGTCGATCACGTGTATTCTG |
| rnf7 | ENSAMXG00000029464 | GTCATGGATGCGTGTCTGA | GACATGCAGCAGTTATGGAAAG |
