# Supplementary material for: Phenotypic plasticity as a mechanism of cave colonization and adaptation
Source: eLife. 2020 Apr 21;9:e51830. doi: 10.7554/eLife.51830 (PMC7173965; doi:10.7554/eLife.51830)
Supplement: Supplementary file 2. [file elife-51830-supp2.docx]

Supplementary File 2. Summary statistics of Illumina output: the number reads, total base pairs, quality trimmed reads retained for each treatment, and the overall mapping rate from Tophat2 using Bowtie2.

| Treatment | Illumina reads | Total Base Pairs | Clean Reads | Overall Mapping Rate (%) |
| --- | --- | --- | --- | --- |
| Dark 1 | 24649247 | 7394774100 | 38834866 | 67.7 |
| Dark 2 | 21923603 | 6577080900 | 33789396 | 68.8 |
| Dark 3 | 24983994 | 7495198200 | 39617512 | 72.9 |
| Light 1 | 27659128 | 8297738400 | 41958430 | 60.0 |
| Light 2 | 23643113 | 7092933900 | 36800490 | 57.6 |
| Light 3 | 25549410 | 7664823000 | 39436392 | 61.8 |
